# Phenotype onset in Huntington’s disease knock‐in mice is correlated with the incomplete splicing of the mutant huntingtin gene

**DOI:** 10.1002/jnr.24493

**Published:** 2019-07-07

**Authors:** Nicholas R. Franich, Miriam A. Hickey, Chunni Zhu, Georgina F. Osborne, Nadira Ali, Tiffany Chu, Nicholas H. Bove, Vincent Lemesre, Renata P. Lerner, Scott O. Zeitlin, David Howland, Andreas Neueder, Christian Landles, Gillian P. Bates, Marie‐Francoise Chesselet

**Affiliations:** ^1^ Department of Neurology, David Geffen School of Medicine University of California, Los Angeles Los Angeles California; ^2^ Department of Pharmacology University of Tartu Tartu Estonia; ^3^ Huntington’s Disease Centre, Department of Neurodegenerative Disease, Queen Square Institute of Neurology University College London London UK; ^4^ UK Dementia Research Institute at UCL University College London London UK; ^5^ Department of Neuroscience University of Virginia School of Medicine Charlottesville Virginia; ^6^ CHDI Management/CHDI Foundation Inc. New York New York

**Keywords:** huntingtin, huntingtin aggregation, huntingtin splicing, mouse behavior, mouse models, neurodegenerative disease, pathology, polyglutamine, RRID:AB_528290, RRID:AB_528297, RRID:AB_532270

## Abstract

Huntington’s disease (HD) is a progressive neurodegenerative disorder caused by an expanded CAG repeat within the huntingtin (*HTT*) gene. The Q140 and *Hdh*Q150 knock‐in HD mouse models were generated such that *Hdh*Q150 mice have an expanded CAG repeat inserted into the mouse *Htt* gene, whereas in the Q140s, mouse exon 1 *Htt* was replaced with a mutated version of human exon 1. By standardizing mouse strain background, breeding to homozygosity and employing sensitive behavioral tests, we demonstrate that the onset of behavioral phenotypes occurs earlier in the Q140 than the *Hdh*Q150 knock‐in mouse models and that huntingtin (HTT) aggregation appears earlier in the striata of Q140 mice. We have previously found that the incomplete splicing of mutant *HTT* from exon 1 to exon 2 results in the production of a small polyadenylated transcript that encodes the highly pathogenic mutant HTT exon 1 protein. In this report, we have identified a functional consequence of the sequence differences between these two models at the RNA level, in that the level of incomplete splicing, and of the mutant exon 1 HTT protein, are greater in the brains of Q140 mice. While differences in the human and mouse exon 1 HTT proteins (e.g., proline rich sequences) could also contribute to the phenotypic differences, our data indicate that the incomplete splicing of *HTT* and approaches to lower the levels of the exon 1 *HTT* transcript should be pursued as therapeutic targets.

## Graphical abstract

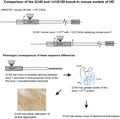

**Significance**

Knock‐in mouse models most accurately reflect the genetic basis of Huntington’s disease, but have been generated by different genetic modification strategies. Here we show that the onset of behavioral phenotypes and the striatal deposition of HTT aggregates occurs earlier in the Q140 than in the *Hdh*Q150 model. This correlated to increased levels of the incompletely spliced *Htt*exon1 transcript and the highly aggregation prone and pathogenic exon 1 HTT protein that it encodes. Our results highlight the importance of developing therapeutic strategies to lower levels of exon 1 HTT.

## 1 INTRODUCTION

Huntington’s disease (HD) is an autosomal dominant, progressive, neurodegenerative disorder caused by a CAG repeat expansion within exon 1 of the huntingtin gene (*HTT*), that encodes a polyglutamine (polyQ) tract in the HTT protein (Bates et al., 2015). Overt symptoms typically appear in midlife and include psychiatric symptoms, cognitive deficits, chorea, and other movement disorders, sleep disturbances and weight loss. HD patients develop characteristic neuropathology involving whole brain atrophy, loss of neurons in the striatum (Vonsattel & DiFiglia, 1998), cortex and other brain regions, and the deposition of HTT inclusions (DiFiglia et al., 1997; Gutekunst et al., 1999). Therapeutic interventions that modify the devastating and fatal course of this disease have yet to be developed.

Mouse models of HD have provided invaluable insights into the pathogenic basis of this disease. Transgenic mice that express an N‐terminal fragment of human *HTT* (Mangiarini et al., 1996; Schilling et al., 1999) develop HD‐related phenotypes with an early onset that progresses rapidly and leads to a considerably shortened lifespan. In contrast, knock‐in models, in which the HD mutation has been introduced into the mouse *Htt* gene, more accurately represent the genetic basis of HD with the mutation being expressed in its appropriate genomic and protein context (Menalled, 2005). The analysis of two HD knock‐in models suggests that sequence differences at the genetically altered loci might modify the age of onset and progression of HD‐related phenotypes *in vivo*. The *Hdh*Q150 line was created by replacing the mouse (CAG)_2_CAA(CAG)_4_ sequence with an expanded CAG repeat, without any other modifications to the locus (Lin et al., 2001). In contrast, in the Q140 line, exon 1 of mouse *Htt* was replaced with exon 1 of human *HTT* carrying an expanded CAG repeat (Hickey et al., 2008; Menalled, Sison, Dragatsis, Zeitlin, & Chesselet, 2003). The Q140 and *Hdh*Q150 mice were generated by genetically modifying 129/Sv and 129/Ola embryonic stem cells, respectively, injecting these into C57BL/6J blastocysts (Lin et al., 2001; Menalled et al., 2003) and backcrossing to C57BL/6J, to produce mice with a mixed strain background.

Robust behavioral deficits have been detected in homozygous Q140 mice on a mixed strain background from as early as 1 month of age (Dorner, Miller, Barton, Brock, & Rebec, 2007; Hickey et al., 2008; Menalled et al., 2003; Rising et al., 2011). A comprehensive analysis of the *Hdh*Q150 mice using the same tests has not been performed, however, the available data indicate that behavioral phenotypes present much later (Brooks, Higgs, Jones, & Dunnett, 2012; Heng, Tallaksen‐Greene, Detloff, & Albin, 2007; Lin et al., 2001; Woodman et al., 2007). These data suggest that sequence differences in the Q140 and *Hdh*Q150 mutant *Htt* genes result in considerable differences in the onset of behavioral deficits. However, comparison of the phenotypes of these mice has been confounded by the fact that they were analyzed on different strain backgrounds and in different laboratories, where husbandry practices and the behavioral tests used had not been standardized. Importantly, the most sensitive tests used to detect early behavioral deficits in the Q140 line have not been used for *Hdh*Q150 mice.

We reasoned that if differences in the onset of behavioral, neuropathological, and molecular deficits could be identified, this might uncover factors that modify the onset of HD symptoms and identify therapeutic targets. In order to directly compare these knock‐in models, we generated Q140 and *Hdh*Q150 C57Bl/6J congenic lines, bred them to homozygosity, and systematically tested them in parallel using identical, sensitive assays of behavioral, pathological and molecular phenotypes, performed at the same ages within the same laboratory. We show that behavioral deficits occur earlier in Q140 as compared to *Hdh*Q150 mice and that this is associated with an earlier evidence of HTT aggregation in the brain. We also show increased levels of the incompletely spliced *Httexon1* transcript that encodes the highly pathogenic exon 1 HTT protein (Neueder et al., 2017; Sathasivam et al., 2013) in Q140 as compared to *Hdh*Q150 C57BL/6J congenic lines. Our work suggests that increased production of the mutant exon 1 HTT protein in Q140 mice may contribute to an earlier phenotype onset and supports the design of therapeutics that decrease the levels of the small *Httexon1* transcript.

## 2 MATERIALS AND METHODS

### 2.1 Animals

At UCLA, all procedures on mice were carried out in accordance with the NIH Guide for the Care and Use of Laboratory Animals (NIH Publications No. 80‐23) revised 1996, and approved by the UCLA Institutional Animal Care and Use Committee. In the United Kingdom, all animal care and procedures were performed in compliance with United Kingdom Home Office regulations (Animals and Scientific Procedures Act 1986) and were approved by the University Ethical Review Process Committee.

#### 2.1.1 Q140 and *Hdh*Q150 knock‐in mice

Homozygous Q140 and *Hdh*Q150 knock‐in mice were backcrossed to C57Bl/6J mice (JAX Stock# 000664) for ten successive generations to generate >99.9% genetically pure C57Bl/6J congenic lines, which was confirmed by single nucleotide polymorphism (SNP) analysis. Mice were bred in animal facilities operated by UCLA Division of Laboratory Animal Medicine (DLAM). For each experiment, mice in all experimental groups were housed under identical conditions. Mice not undergoing behavioral testing were housed in the DLAM facility, under standard light cycle conditions (12 hr on/off, with lights off at 6 p.m.), where they were group housed (2–4 mice per cage), with the exception of some older mice (typically ~12 months) that were singly housed as a result of attrition of cage mates. Mice were fed a diet of standard mouse chow (Harlan‐Teklad) and DLAM facility cages had corncob bedding and several cotton nestlets per cage. All mice that were used for behavioral analysis were transferred from the UCLA DLAM facility to a reverse light cycle facility (12 hr on/off, with lights off at 10 a.m.), 1 week before behavioral testing began. Here, they were group housed (2–4 mice per cage), with standard bedding consisting of shredded wood chips, and material for nest‐building (Nestlets, Ancare, NY, USA), and had ad libitum access to water and standard rodent diet (NIH‐31 modified 7013, Harlan). Room temperature in both mouse facilities was 23°C ± 2°C and relative humidity was ~60%; these values being recorded during a daily animal check. Behavioral testing was performed between 1.00 and 5.00 p.m. during the dark phase, under red light, in a specified room for behavioral experiments adjacent to the animal housing room.

Tail tips were biopsied for genotyping between 14 and 21 days of age and mice were weaned by 21 days of age. Q140 mice genotyping was performed as described in Hickey et al. (2008) and *Hdh*Q150 mice genotyping was performed as described in Heng et al. (2007). Tail biopsies were again collected when mice were euthanized for re‐genotyping. Tail DNA from a subset of mice was used for CAG repeat length analysis at Laragen, Inc. (Culver City, CA). For Q140 homozygotes (*n* = 18), the longer allele had a mean of 127.2 ± 9.0 (*SD*) CAGs, while the shorter allele had a mean of 119.2 ± 4.6 (*SD*) CAGs. For *Hdh*Q150 homozygotes (*n* = 17), the longer allele had a mean of 141.1 ± 8.0 (*SD*) CAGs, while the shorter allele had a mean of 128.4 ± 8.0 (*SD*) CAGs. Therefore, the *Hdh*Q150 mice had significantly longer CAG repeat lengths than Q140 mice (two‐tailed Student’s *t* tests, larger allele: *p* = 0.00004, smaller allele: *p* = 0.0001).

For each experiment, all mice in each experimental group were euthanized and tissue collected at the same time of day. Mice used for behavioral analysis were euthanized during the dark phase of the diurnal cycle, 1–3 hr after lights off. All other mice were euthanized during the light phase of the diurnal cycle, 5–8 hr after lights on.

#### 2.1.2 zQ175 and zQ175DN knock‐in mice

The zQ175 knock‐in line arose through a spontaneous expansion of the CAG repeat in line Q140 (Heikkinen et al., 2012; Menalled et al., 2012) and both the Q140 and zQ175 lines retained a neomycin resistance gene cassette located 1.3 kb upstream of the ATG. In order to test whether this might affect the expression of *Htt*, or the level of incomplete splicing, we utilized zQ175 mice, from the CHDI Foundation colony at the Jackson Laboratories (Bar Harbor, Maine) in which the neomycin cassette had been removed. Heterozygous zQ175 females (JAX Stock# 370476) had been crossed to homozygous male C57BL/6‐Tg(Zp3‐cre)93Knw/J (JAX Stock# 003651) in which Cre was expressed under the control of the zona pellucida (Zp3) promoter (de Vries et al., 2000), which drives expression exclusively in growing oocytes before completion of the first meiotic division. The resultant zQ175DN (delta *neo*) colony, on a C57BL/6J background (JAX Stock# 000664) was established. zQ175, zQ175DN, and WT mice were transferred to the Bates colony in London where they were euthanized at 2 months of age, brain regions were snap frozen in liquid nitrogen and stored at −80°C.

### 2.2 Cohorts of mice used for behavioral testing

The number of animals used was based on previous experiments showing robust behavioral deficits in homozygous Q140 mice on a mixed strain background from as early as 1 month of age (Dorner et al., 2007; Hickey et al., 2008; Menalled et al., 2003; Rising et al., 2011).

#### 2.2.1 Cohorts of mice used for the open field, Y‐maze, and pole task (1–6 months of age)

All Q140, *Hdh*Q150, and their WT littermates were tested on the pole task (at 4 months of age). A subset of these mice was used at 1, 4, and 6 months for open field testing; not all mice that underwent pole task testing were used for open field because genotypes were not available for some mice at 1 month of age. The Y‐maze was introduced as a behavioral test part way through the study, therefore only a subset of mice that were enrolled later in the study was used in this test. The final group sizes for each test were as detailed below. WT = wild type, Mut = mutant. *For open field*: Q140 WT (*n* = 10; male *n* = 3, female *n* = 7), Q140 Mut (*n* = 9; male *n* = 4, female *n* = 5), *Hdh*Q150 WT (*n* = 20; male *n* = 10, female *n* = 10), *Hdh*Q150 Mut (*n* = 20; male *n* = 12, female *n* = 8). *For Y‐maze testing*: Q140 WT (*n* = 7; male *n* = 3, female *n* = 4), Q140 Mut (*n* = 5; male *n* = 2, female *n* = 3), *Hdh*Q150 WT (*n* = 14; male *n* = 7, female *n* = 7), *Hdh*Q150 Mut (*n* = 10; male *n* = 6, female *n* = 4). *For the pole task*: Q140 WT (*n* = 10; male *n* = 6, female *n* = 4), Q140 Mut (*n* = 9; male *n* = 4, female *n* = 5), *Hdh*Q150 WT (*n* = 29; male *n* = 13, female *n* = 16), *Hdh*Q150 Mut (*n* = 24; male *n* = 15, female *n* = 9). For all tests, the proportion of males and females did not significantly differ between groups. Fisher’s exact tests: open field, Q140 WT v. Q140 Mut *p* = 0.7512, *Hdh*Q150 WT v. *Hdh*Q150 Mut, *p* = 0.6499; Y‐maze, Q140 WT v. Q140 Mut *p* = 1.000, *Hdh*Q150 WT v. *Hdh*Q150 Mut, *p* = 0.6968; pole task, Q140 WT v. Q140 Mut *p* = 0.6563, *Hdh*Q150 WT v. *Hdh*Q150 Mut, *p* = 0.2712.

#### 2.2.2 Cohorts of mice used for the running wheel test at 6 months of age

A separate cohort of 6 month old Q140, *Hdh*Q150, and WT male mice was used for running wheel analysis. Only male mice were used because running wheel performance is affected by gender and the estrous cycle (Hickey et al., 2008). Group sizes were as follows: Q140 WT (*n* = 6), Q140 Mut (*n* = 6), *Hdh*Q150 WT (*n* = 14), and *Hdh*Q150 Mut (*n* = 10). Two additional Q140 WT mice were included in running wheel testing, but equipment failed to detect wheel rotations for the duration of testing for these two mice. At the conclusion of the 2 weeks of testing, fresh frozen brain tissue was collected from all mice that were included in running wheel testing at 6.5 months of age for RNA analysis (see below).

### 2.3 Behavioral testing

WT and homozygous Q140 and *Hdh*Q150 mice used for behavioral testing were habituated to housing rooms adjacent to our laboratory’s behavioral facility for 1 week prior to the tests commencing. Mice were housed with littermates (up to 4 per cage) in reverse light–dark cycle conditions (10 a.m. to 10 p.m.). All tests were performed and analyzed by an experimenter blind to genotype.

#### 2.3.1 Open field (1, 4 and 6 months of age)

Behavior in an open field apparatus (Tru Scan, Colbourn, Allentown PA) was conducted under red lighting starting in the dark phase 1 hr after lights off and finishing at the end of the fourth hour after lights off. The open field apparatus consists of four clear plexiglass walls (26.5 by 37.5 cm) that form an enclosure with two rows of 16 infrared photocell beams to measure total horizontal locomotor activity. Mice were placed into the open field and activity was recorded for 15 min. The apparatus was cleaned using Conflikt (Decon Laboratories, King of Prussia, PA) to remove odors between trials.

#### 2.3.2 Spontaneous alternation in the Y‐maze (3 months of age)

The Y‐maze was used to test spatial working memory (Magen et al., 2012). The apparatus was a three‐arm horizontal maze in which the arms are symmetrically disposed at 120° angles to each other. Two arms (B and C) were 15 cm in length and one arm (A) was 20 cm long. All arms were 5 cm in width and the walls were 12 cm high. The maze was made out of opaque white plastic boards and attached to a square plastic floor 27 cm × 27 cm. Mice were habituated to the room for 1 hr prior to the test, in the dark phase. The maze was placed in a red light illuminated room. Mice were initially placed in the long arm (A) with their head facing the maze arms (toward the point at which arms meet). The animals were given 7 min to explore the maze, while a tripod mounted video camera recorded arm entries. The experimenter could not be seen by the animal during this time. After 7 min, the animal was removed from the maze and returned to its home cage. The maze was thoroughly cleaned with 70% of ethanol and dried after each trial. Alternation in the Y‐maze was defined as consecutive entry into all three arms (e.g., ABC, CAB, or BCA, but not BAB). Behavior was video‐recorded and alternations were counted for all of the overlapping sequences of three consecutive entries by an investigator blind to the experimental groups. The fraction of alternations is defined as follows: [(number of alternations)/(total number of arm entries−2)].

#### 2.3.3 Pole task (4 months of age)

Mice were examined at 4 months of age for motor performance on a vertical pole (1 cm in diameter, 60 cm high) as described previously (Hickey et al., 2008). In this test, mice were placed at the top of a vertical pole facing upwards, they need to turn to face downwards and descend the pole. Mice were habituated to the task in two trials per day for 2 days. On the test day (day 3) three measures were taken over five trials per mouse: the total time taken to turn and descend, the time taken to turn and the time taken to descend after turning.

#### 2.3.4 Running wheel (6–6.5 months of age)

Mice were placed individually in cages equipped with a running wheel (23 cm diameter, Mini Mitter Company Inc., Bend, OR). Each wheel rotation was recorded in 3‐min bins by VitalView Data Acquisition Software V 4.0 (Mini Mitter Company Inc.). Running wheel cages were housed in cabinets (eight cages/cabinet) to minimize light and sound disturbance (lights off 10 a.m., lights on 10 a.m., synchronized with the lights in the housing room). Cabinets were equipped with fans to allow air circulation. Running activity was recorded continuously for 14 days. After 7 days mice were removed from the cages during the light phase, weighed and their food, water and bedding changed. Wheel running activity during light and dark phases was calculated using ActiView V 1.2 (Mini Mitter Company Inc.). The level of activity during each dark phase (“night”) was calculated and used to generate the mean number of rotations per 3 min for each night.

### 2.4 Antibodies

The antibodies that were used for immunohistochemistry, immunoprecipitation and western blotting are summarized in Table 1. 3B5H10 (RRID:AB_532270) is a monoclonal antibody that was raised against an N‐terminal 171 amino acid fragment of HTT with 65Q and detects a polyQ tract (Peters‐Libeu et al., 2005). MW1 (RRID:AB_528290) and MW8 (RRID:AB_528297) are monoclonal antibodies that were raised against soluble and aggregated versions of exon 1 HTT respectively (Ko, Ou, & Patterson, 2001). MW1 and 3B5H10 recognize an expanded polyQ tract and MW8 acts as a neoepitope antibody on western blots that detects the AEEPLHRP peptide at the C‐terminus of exon 1 HTT (Landles et al., 2010). These three antibodies were extensively characterized in Landles et al. (2010), and the results in this paper are consistent with the immunodetection of HTT.

**Table 1:** Summary of the antibodies

Name	Epitope	Species	Concentration	Source
MW1	PolyQ	Mouse monoclonal	WB (1:1,000)	CHDI Foundation
MW8	AEEPLHRP	Mouse monoclonal	WB (1:1,000)	CHDI Foundation
			IHC (1:1,000)	
3B5H10	PolyQ	Mouse monoclonal	IP 2 µg	Sigma P1874

Abbreviations: IHC, immunohistochemistry; IP, immunoprecipitation; WB, western blotting.

### 2.5 HTT immunohistochemistry

Male homozygous Q140 and *Hdh*Q150 mice (*n* = 3/genotype) at 4.5 and 12–13 months of age were perfused transcardially with 0.01M of phosphate buffered saline pH 7.2 (PBS), followed by 4% of paraformaldehyde (PFA), and 0.5% glutaraldehyde in PBS. The brains were removed, post‐fixed in 4% of paraformaldehyde in 0.1M PBS for 6 hr at 4°C, cryoprotected in 30% of sucrose for 72 hr, cut into 35‐µm‐thick sagittal sections on a Leica CM 1800 cryostat (Deerfield, IL) and stored at −20° in a glycerol‐based cryoprotectant solution. Sections were processed for immunostaining with MW8 (Table 1). After washes in 0.01M PBS, the sections were treated with H_2_O_2_ (1%) in PBS containing 0.5% of Triton X‐100 for 20 min to block endogenous peroxidase activity. Nonspecific sites were blocked by incubating the sections for 30 min at room temperature in PBS containing 3% of bovine serum albumin (BSA) and 2% of normal goat serum (NGS). The sections were incubated with MW8 1:1,000 in PBS containing 3% of BSA, 2% of NGS, and 0.2% of Triton X‐100 (incubation buffer) for 24 hr at room temperature. After rinses in PBS, the sections were incubated in biotinylated goat anti‐rabbit antibody (1:200) (Vector ABC Elite, Burlingame, CA) in the incubation buffer for 2 hr at room temperature. After several rinses in PBS, the sections were incubated for 2 hr in avidin‐biotin complex (Vector ABC Elite) in PBS containing 0.2% Triton X‐100. Immunoreactivity was visualized by incubation in 0.03% 3‐3′‐diaminobenzidine tetrahydrochloride (Sigma, St. Louis, MO) and 0.0006% H_2_O_2_ in 0.05 M Tris buffer, pH 7.6. After rinses in cold Tris buffer, the sections were dehydrated, defatted in xylene, and mounted with Eukitt (Calibrated Instruments, Hawthorne, NY). Images of HTT staining were captured using an Olympus microscope connected to a PC with SPOT imaging acquisition software.

### 2.6 Immunoprecipitation and western blotting

Immunoprecipitation with 3B5H10 and mouse IgG (control) followed by western blotting with MW1 and MW8 were performed as previously described (Landles et al., 2010).

### 2.7 Quantitative real‐time PCR analysis of selected striatal transcripts

At the conclusion of running wheel testing, mice were euthanized by cervical dislocation and fresh striatal and cortical tissue was dissected using a coronal brain matrix for mice with 1 mm divisions (Stoelting Co., Wood Dale, IL). Dissected tissue was snap‐frozen in liquid nitrogen and stored at −80º C. Total RNA was purified from fresh‐frozen brain tissue using Qiagen RNeasy mini kit (Qiagen) according to the manufacturers recommendations. During the RNA extraction procedure, on‐column DNase I treatment was performed to remove contaminating genomic DNA (RNase‐Free DNase Set, Qiagen). RNA was stored at −80**°**C. RNA integrity was confirmed using a BioAnalyzer (Agilent RNA 6000 Nano Assay, UCLA Genotyping and Sequencing Core). For cDNA synthesis with random hexamer primers, a High‐Capacity cDNA reverse transcription (RT) kit (Applied Biosystems) was used. cDNA was stored at −20**°**C. The cDNA was then analyzed by quantitative real time PCR (qPCR) using an Applied Biosystems 7900HT Real time PCR machine (UCLA Genotyping and Sequencing Core). PCR reactions were performed using TaqMan gene expression assays (Applied Biosystems) using inventoried primer‐probe sets, with FAM labeled probes. Assay identification numbers of Applied Biosystems inventoried primer‐probe set TaqMan assays of striatal transcripts were: dopamine‐ and cAMP‐regulated phosphoprotein 32 (*Darpp32*), Mm00454892_m1; dopamine receptor D1 (*Drd1a*), Mm01353211_m1; dopamine receptor D2 (*Drd2*), Mm00438545_m1; cannabinoid receptor 1(*Cnr1*), Mm01212171_s1; preproenkephalin (*Penk*), Mm01212875_m1; high‐affinity glutamate transporter 1 (*Glt1*), Mm00441457_m1; substance P receptor (*Tac1*), Mm00436892_m1. Identification numbers of Applied Biosystems inventoried primer‐probe set TaqMan assays of striatal and cortical reference genes were: ATP synthase, H + transporting mitochondrial F1 complex, beta subunit (*Atp5b*), Mm00443967_g1; eukaryotic translation initiation factor 4A2 (*Eif4a2*), Mm00834357_g1; peptidylprolyl isomerase A (*Ppia*), Mm02342429_g1. For each gene, RT + samples from each animal and no‐template controls samples were run in triplicate and RT‐samples run in parallel. The PCR cycling parameters were 50°C for 2 min, 95°C for 10 min (1 cycle); 95°C for 15 s, 60°C for 1 min (40 cycles). Relative expression of genes of interest was determined by normalizing to the geometric mean of the two most stable reference genes identified using the geNorm applet for Microsoft Excel (reference genes are *Atp5b*, *Ppia* and *Eif4a2*), as previously described (Vandesompele et al., 2002).

### 2.8 Analysis of *Htt* transcripts by quantitative real‐time PCR

Fresh striatal, cortical, and cerebellar tissue was dissected from mice at 2 months of age, snap‐frozen in liquid nitrogen and stored at −80ºC. Tissue was homogenized in Qiazol buffer (Qiagen) before total RNA isolation using the QiaGen RNeasy mini kit (Qiagen) and RNA was quantified using a Nanodrop 1000. RNA was reverse transcribed using MMLV‐RT (Invitrogen) and oligo‐dT primers (Invitrogen) and cDNA was stored at −20**°**C. qPCR was performed using SsoFast Probes Supermix (Bio‐Rad) with a corresponding cycler program using a CFX 96 qPCR system (Bio‐Rad). Primer‐probe set gene expression assays for the *Htt* intron 1 sequences were 347F‐431R‐371P and for spliced *Htt* were ‐19F‐ex2R‐ex2P as detailed in Table 2. Housekeeping reference genes were *Atp5b*, *Eif4a2*, and *Ubc* (striatum and cerebellum) and *Atp5b*, *Eif4a2*, *Canx*, and *Rpl13a* (cortex) from Primer Design (Benn, Fox, & Bates, 2008). The level of intronic sequences was determined by normalizing to housekeeping genes. The relative levels of the spliced transcript was determined by the 2*^−ΔΔCt^* method (Benn et al., 2008).

**Table 2:** qPCR assays used to determine comparative levels of *Htt* transcripts

Assay	Primer/Probe	Sequence
Intron 1: 347F‐431R‐371P	Forward	5′‐TCCTCATCAGGCCTAAGAGCTGG‐3′
	Reverse	5′‐GAGACCTCCTAAAAGCATTATGTCATC‐3′
	Probe	5′‐AGTGCAGGACAGCGTGAGAGATGTG‐3′
Exon 1—Exon 2‐19F‐ex2R‐ex2P	Forward	5′‐AGGAACCGCTGCACCGA‐3′
	Reverse	5′‐CTGAGAGACTGTGCCACAATGTT‐3′
	Probe	5′‐AGAAAGACCGTGTGAATCATTGTCTAACAATATGTGA‐3′

Note

The primers and probes for the TaqMan qPCR assay are listed.

### 2.9 Statistical analysis

A critical value of *p* < 0.05 was used for all statistical analysis. Grubb’s test for outliers was performed on data from each treatment group and if a significant outlier was identified in a group, it was removed before comparisons between groups were performed. SigmaPlot v12 (Systat) or SPSS (v22) was used for all statistical analysis using one‐way ANOVA, two‐way ANOVA, or repeated‐measures two‐way ANOVA, as appropriate, with Bonferroni or Fisher’s LSD post hoc tests, as indicated. Planned comparison Student’s *t* tests were also used where indicated. Graphs were prepared using GraphPad Prism.

## 3 RESULTS

To evaluate whether phenotypes develop at different rates in the Q140 and *Hdh*Q150 KI mice, we began by standardizing the genetic background. Q140 and *Hdh*Q150 mice were backcrossed to C57BL/6J for 10 successive generations to generate 99.9% genetically pure congenic lines that were then bred to homozygosity. CAG repeat sizes were relatively comparable between the lines but longer in the *Hdh*Q150 (141.1 ± 8.0 and 128.4 ± 8.0) compared to the Q140 (127.2 ± 9.0 and 119.2 ± 4.6) mice.

### 3.1 Q140 homozygotes, but not *Hdh*Q150 homozygotes are impaired in behavioral tasks before 6 months of age

#### 3.1.1 Exploratory activity in the open field

**Figure 1:**
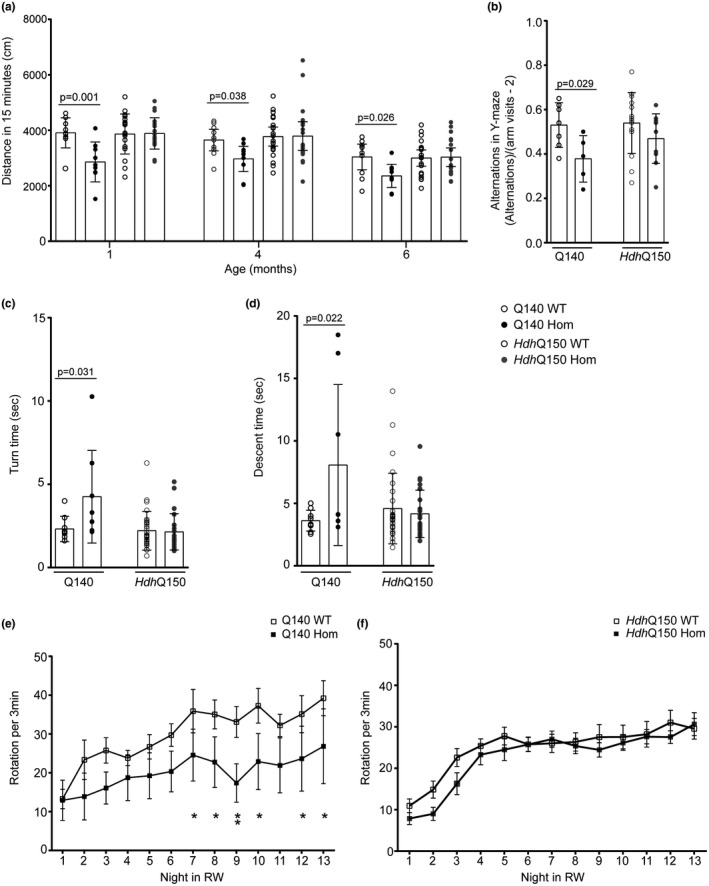
Behavioral characterization of homozygous C57BL/6J congenic Q140 and *Hdh*Q150 mice. (a) Total movement in open field for homozygous Q140 and *Hdh*Q150 mice and WT littermates. Graph shows mean ± *SEM* distance (cm) during 15 min in the open field apparatus at 1, 4, and 6 months of age. Two‐way repeated measures ANOVA with Fisher’s LSD post hoc tests at each age. Group sizes: Q140 mutant (*n* = 9), Q140 WT littermates (*n* = 10), *Hdh*Q150 mutant (*n* = 20), *Hdh*Q150 WT littermates (*n* = 20). (b) Spontaneous alternation in Y‐maze of homozygous Q140 and *Hdh*Q150 mice and WT littermates at 3 months of age. Graph shows mean ± *SEM* fraction of alternations calculated as number of sequential entries into all three arms of the Y‐maze, divided by the total number of arm visits minus 2. Two‐tailed Student’s *t* test. Group sizes: Q140 mutant (*n* = 5), Q140 WT littermates (*n* = 7), *Hdh*Q150 mutant (*n* = 10), *Hdh*Q150 WT littermates (*n* = 14). (c and d) Time to turn and descend a vertical pole for homozygous Q140 and *Hdh*Q150 mice and their WT littermates at 4 months of age. Graphs show mean ± *SEM* time (sec) to turn (c) and descend (d) a vertical pole. One‐way ANOVA with Bonferroni post hoc tests. Group sizes: Q140 mutant (*n* = 8), Q140 WT littermates (*n* = 9), *Hdh*Q150 mutant (*n* = 23), *Hdh*Q150 WT littermates (*n* = 28). (e and f) Running wheel performance in homozygous Q140 and *Hdh*Q150 mice and their WT littermates at 6 months of age. Graphs show mean ± *SEM* running wheel (RW) rotations per 3 min, during the 12‐hr dark phase of the 24‐hr light cycle (night). Graphs show performance of 6 month old male mice: (e) Q140 mutant mice and WT littermates, (f) *Hdh*Q150 mutant mice and WT littermates, during each night over two weeks housed in the RW apparatus. Repeated measures two‐way ANOVA (comparing all four genotype groups; graphs show knock‐in mouse lines separately for clarity). Fisher’s LSD post hoc comparisons showed no difference in running wheel activity on nights 1 (*p* = 0.417), 2 (*p* = 0.051), 3 (*p* = 0.089), 4 (*p* = 0.371), 5 (*p* = 0.190), 6 (*p* = 0.100), or 11 (*p* = 0.071), but significantly reduced running was observed on nights 7 (*p* = 0.048), 8 (*p* = 0.032), 9 (*p* = 0.007), 10 (*p* = 0.013), 12 (*p* = 0.045), and 13 (*p* = 0.031)_,_ There was no difference detected by Fisher’s LSD post hoc comparisons for the *Hdh*Q150 mice (nights 1 (*p* = 0.450), 2 (*p* = 0.151), 3 (*p* = 0.163), 4 (*p* = 0.701), 5 (*p* = 0.419), 6 (*p* = 0.993), 7 (*p* = 0.804), 8 (*p* = 0.809), 9 (*p* = 0.447), 10 (*p* = 0.728), 11 (*p* = 0.883), 12 (*p* = 0.388), and 13 (*p* = 0.800). Group sizes: Q140 mutant (*n* = 6), Q140 WT littermates (*n* = 6), *Hdh*Q150 mutant (*n* = 10), *Hdh*Q150 WT littermates (*n* = 14)

Spontaneous locomotion of homozygous Q140 and *Hdh*Q150 mice, together with the WT littermates from both lines was tested in an open field apparatus at 1, 4 and 6 months of age (Figure 1a). At all ages, Q140 mice showed a reduction in exploratory behavior, with a shorter total movement distance during 15 min in the open field [two‐way repeated measures ANOVA, *F*(genotype)_3,106_ = 6.419, *p* = <0.001; *F*(age)_2,106_ = 28.581, *p* < 0.001; *F*(age × genotype)_6,106_ = 0.251, *p* = 0.958. Fisher’s LSD post hoc comparisons at each age: 1 month Q140 MUT v WT, *p* = 0.001; 4 month Q140 MUT v WT, *p* = 0.038; 6 month Q140 MUT v WT, *p* = 0.026]. In contrast, *Hdh*Q150 mice showed normal exploration in the open field at all of these ages [Fisher’s LSD post hoc comparisons at each age: 1 month *Hdh*Q150 MUT v WT, *p* = 0.923; 4 month *Hdh*Q150 MUT v WT, *p* = 0.842; 6 month *Hdh*Q150 MUT v WT, *p* = 0.693].

#### 3.1.2 Spontaneous alternation in a Y‐maze

Spatial working memory was tested by spontaneous alternation in a Y‐maze in 3 month old mice (Figure 1b). Q140 mice showed a reduced spontaneous alternation compared to WT littermates [two‐tailed Student’s *t* test, *p* = 0.029], whereas the performance of *Hdh*Q150 mice was not significantly different from their WT counterparts at the same age [two‐tailed Student’s *t* test, *p* = 0.197]. Therefore, spatial working memory becomes impaired earlier in Q140 mice.

#### 3.1.3 Descent from a vertical pole

Mice were placed at the top of a vertical pole in a head‐up orientation. At 4 months of age, Q140 mice showed an impairment both in the time to turn to a head‐down position, as well as in the time taken to descend the pole, as compared to their WT littermates [One‐way ANOVA: time to turn, *F*(genotype)_3,64_ = 5.254, *p* = 0.003; Bonferroni post hoc comparisons: Q140 MUT v WT, *p* = 0.031; time to descend, *F*(genotype)_3,64_ = 3.917, *p* = 0.012; Bonferroni post hoc comparisons: Q140 MUT v WT, *p* = 0.022]. In contrast, *Hdh*Q150 mice were not impaired at this task [Bonferroni post hoc comparisons: time to turn, *Hdh*Q150 MUT v WT, *p* = 1.000; time to descend, *Hdh*Q150 MUT v WT, *p* = 1.000] (Figure 1c,d).

#### 3.1.4 Performance in a running wheel

Running wheel performance in a separate cohort of male Q140 mice and *Hdh*Q150 mice, as compared to their WT littermates, was tested at 6 months of age. Q140 mice showed a reduced mean nightly running wheel performance compared to WT littermates. Two‐way repeated measures ANOVA: *F*(genotype)_3,372_ = 2.035, *p* = 0.129, *F*(night)_12,372_ = 23.795, *p* < 0.001, *F*(genotype × night)_36,372_ = 0.986, *p* = 0.496. Fisher’s LSD post hoc comparisons showed significantly reduced running on nights 7 (*p* = 0.048), 8 (*p* = 0.032), 9 (*p* = 0.007), 10 (*p* = 0.013), 12 (*p* = 0.045) and 13 (*p* = 0.031). There was no difference detected by Fisher’s LSD post hoc comparisons for the *Hdh*Q150 mice (Figure 1e,f).

In summary, homozygous Q140 have decreased locomotion in the open field at 1–6 months, decreased spontaneous alternation in the Y‐maze at 3 months, impairment in the pole task at 4 months and decreased running wheel performance at 6 months. We did not detect any deficits in the homozygous *Hdh*Q150 mice in these tests at the same ages.

### 3.2 HTT immunostaining detects aggregates in the striatum earlier in Q140 than in *Hdh*Q150 mice

The choice of antibody for use in immunohistochemical experiments to investigate the timing of HTT deposition in the brains of these two mouse models had to be considered carefully. The Q140 and *Hdh*Q150 mice express human and mouse exon 1 HTT, respectively, and many antibodies raised against exon 1 HTT sequences, detect human, and mouse HTT differently. For example, monoclonal EM48 does not detect mouse HTT (Wang et al., 2008) and therefore polyclonal EM48 may detect human and mouse HTT with different affinities. Similarly, the polyclonal S830 (Sathasivam et al., 2001) most likely recognizes the human proline rich sequence and also should not be used. In contrast, the epitope for MW8 is present in both mouse lines (Ko et al., 2001), this antibody readily detects aggregated HTT (Ko et al., 2001; Landles et al., 2010), and we have no evidence to indicate that this antibody has a different affinity for human and mouse HTT.

**Figure 2:**
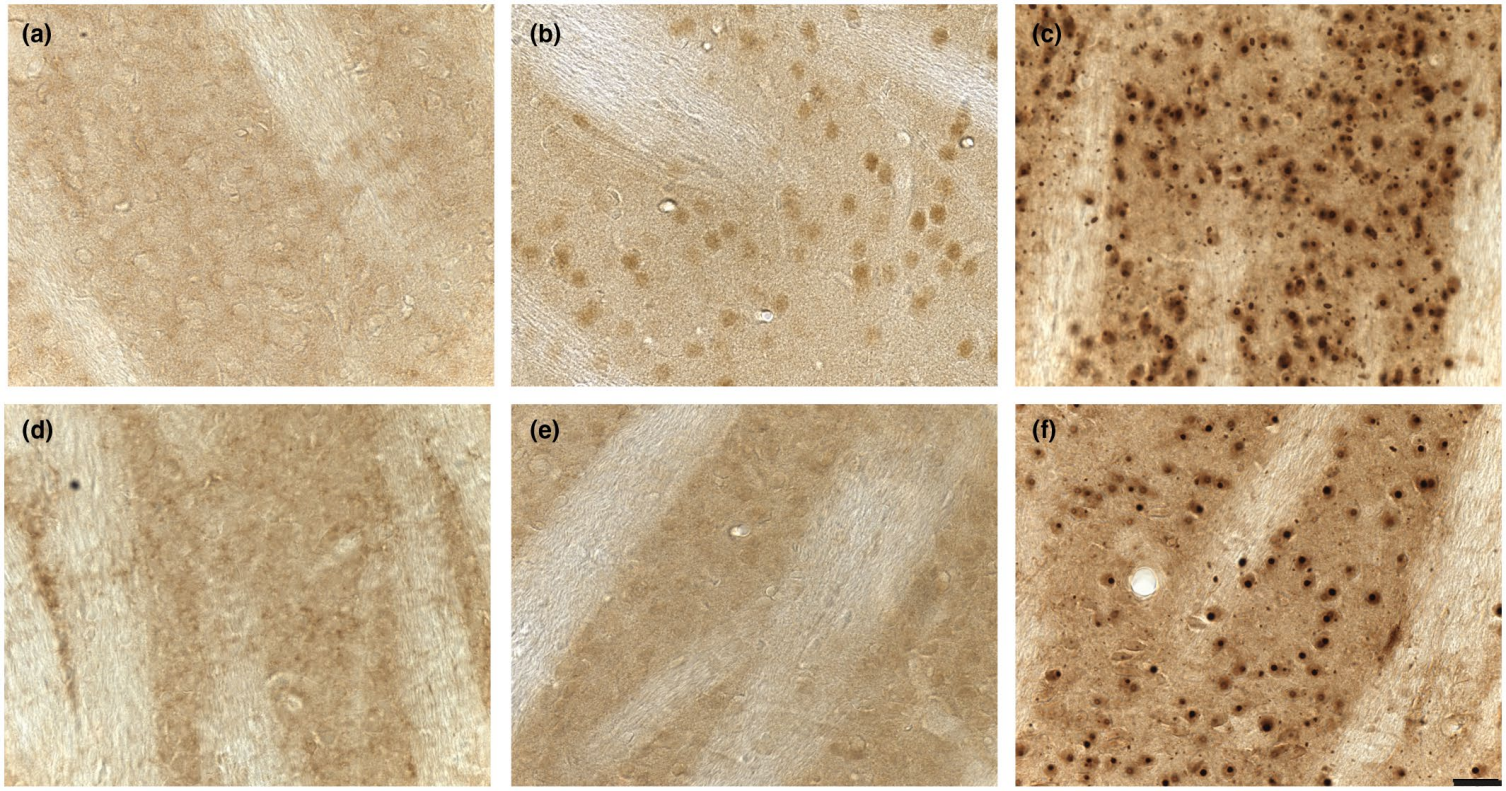
Striatal HTT immunostaining in homozygous Q140 and *Hdh*Q150 mice. Sagittal striatal sections from (a) WT mice aged 7 months, (b) Q140 mice aged 4.5 months (c) Q140 mice aged 13 months, (d) WT mice aged 12 months, (e) *Hdh*Q150 mice aged 4.5 months and (f)) *Hdh*Q150 mice aged 12 months immunostained with MW8. At 4.5 months of age, Q140 mice showed stronger diffuse nuclear HTT staining compared with *Hdh*Q150 mice. At 12–13 months of age, both Q140 and *Hdh*Q150 mice have HTT inclusions and neuropil aggregates. However, Q140 mice show more neuropil aggregates in the striatum than *Hdh*Q150 mice. Scale bar = 20 μm, applies to all images

Striatal sections from homozygous Q140 and *Hdh*Q150 mice aged 4.5 and 12–13 months and their WT controls were immunoprobed with MW8. No specific staining was observed in the brains of the WT mice at 7 and 12 months, respectively (Figure 2a,d). At 4.5 months of age, a diffusely distributed HTT species had accumulated in neuronal nuclei in the striatum of Q140 mice and small nuclear inclusions could be observed in some sections (Figure 2b). In contrast, this nuclear accumulation of HTT was barely detectable on sections from *Hdh*Q150 animals (Figure 2e). At 12–13 months of age, the nuclear inclusions and neuropil aggregates were readily observed in both Q140 and *Hdh*Q150 striata (Figure 2c,f), and the Q140 mice appeared to have smaller nuclear inclusions and a greater number of neuropil aggregates.

### 3.3 Homozygous Q140 and *Hdh*Q150 mice have similar levels of transcriptional dysregulation in the striatum at 6.5 months of age

Transcriptional dysregulation is a well‐defined molecular HD phenotype that has been extensively studied in HD mouse models and in *post mortem* brains (Hodges et al., 2006; Langfelder et al., 2016), for which distinct changes in the expression pattern of striatal genes has been documented. To determine whether differences between Q140 and *Hdh*Q150 mice could be observed at the level of transcriptional changes, we measured the levels of selected striatal transcripts by real time quantitative PCR (qPCR) in male homozygous Q140 and *Hdh*Q150 mice at 6.5 months of age. Brains had been harvested from these mice immediately following the completion of running wheel behavioral testing in which the performance of Q140 mice, but not *Hdh*Q150 mice, was impaired.

**Figure 3:**
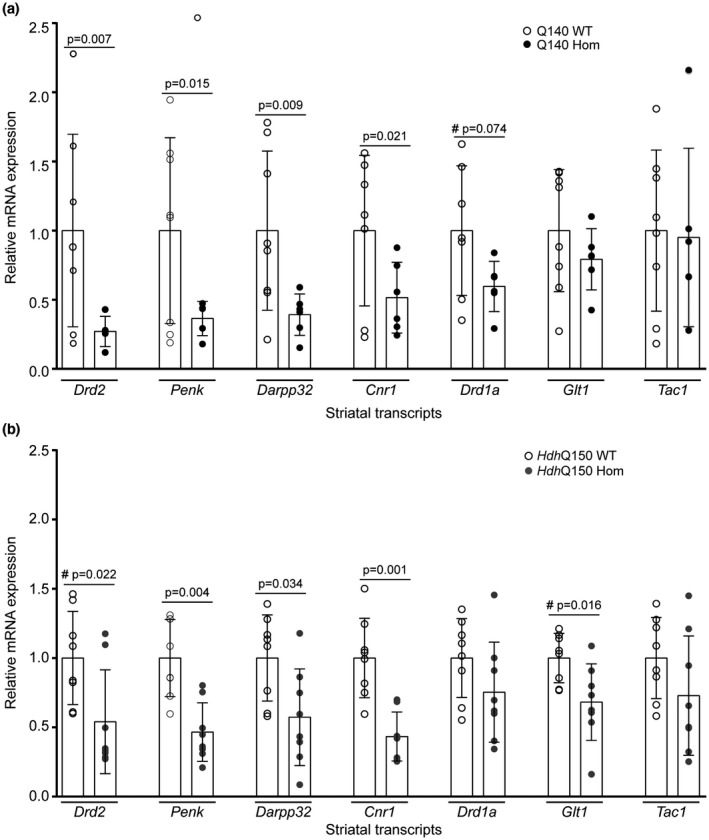
Expression levels of striatal transcripts in homozygous Q140 and *Hdh*Q150 mice and their WT littermates at 6.5 months of age. Relative levels of selected striatal transcripts assessed by qPCR in 6.5‐month–old‐male (a) homozygous Q140 mice (*n* = 5–6) and WT littermates (*n* = 7–8) and (b) homozygous *Hdh*Q150 mice (*n* = 8) and WT littermates (*n* = 8). One‐way ANOVA comparing all four genotypes with Fisher’s LSD post hoc tests, mean + *SEM*: *Drd2*: *F*(genotype)_3,25_ = 3.990, *p* = 0.019; *Penk*: *F*(genotype)_3,25_ = 5.917, *p* = 0.003; *Darpp32*: *F*(genotype)_3,26_ = 4.463, *p* = 0.012; *Cnr1*: *F*(genotype)_3,25_ = 6.526, *p* = 0.002; *Drd1a*: *F*(genotype)_3,25_ = 2.338, *p* = 0.098; *Glt1*: *F*(genotype)_3,26_ = 2.388, *p* = 0.092; *Tac1*: *F*(genotype)_3,26_ = 0.519, *p* = 0.673. #Planned comparison two‐tailed Student’s *t* tests. *Drd2* = dopamine receptor D2, *Penk1* = preproenkephalin, *Darpp32* = dopamine‐ and cAMP‐regulated phosphoprotein 32, *Cnr1* = cannabinoid receptor 1, *Glt1* = glutamate transporter, *Tac1* = tachykinin 1 receptor (Substance P receptor)

The expression levels of the striatal genes: dopamine receptor D2 (*Drd2*), preproenkephalin (*Penk1*), *Darpp32*, and cannabinoid receptor 1 (*Cnr1*) mRNAs were decreased in both Q140 and *Hdh*Q150 mice (Figure 3a,b). This is consistent with our previous data for 4.5 month old homozygous Q140 mice on a mixed strain background (Hickey, Franich, Medvedeva, & Chesselet, 2011). Therefore, the Q140 and *Hdh*Q150 mice have similar decreases in the expression levels of these signature striatal genes at 6.5 months, despite the absence of motor phenotypes in the *Hdh*Q150 model, and its better performance on the running wheel task (which might be expected to increase transcription levels). Given that this transcriptional dysregulation precedes the onset of behavioral deficits in the *Hdh*Q150 mice, the transcriptional repression of *Drd2*, *Penk1*, *Darpp32*, or *Cnr1* does not influence the onset of the behavioral phenotypes that we have measured in these knock‐in lines.

### 3.4 The incomplete splicing of *Htt* is greater in the Q140 than *Hdh*Q150 mice

We have previously shown that exon 1 of *HTT* does not always splice to exon 2 generating a small exon 1—intron 1 polyadenylated mRNA (*Httexon1*) that encodes the highly pathogenic exon 1 HTT protein. Termination of this mRNA occurs at cryptic polyadenylation sites located at 680 and 1,145 bp into intron 1. This level of incomplete splicing increases with increasing CAG repeat length and occurs in all knock‐in mouse models of HD, in YAC128 mice, and in HD postmortem brains and fibroblast cell lines (Neueder et al., 2017; Sathasivam et al., 2013). Therefore, we set out to determine whether levels of incomplete splicing, and consequently, of the exon 1 HTT protein, might underlie the differences in the onset of HD‐related phenotypes in these mouse models.

**Figure 4:**
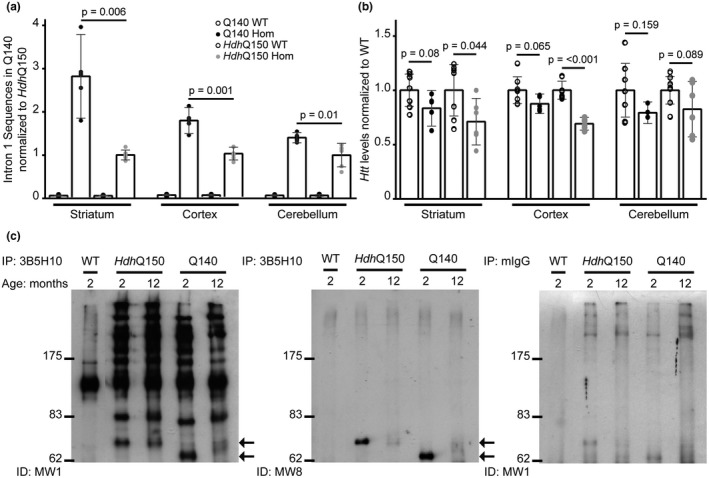
The level of the *Httexon1* transcript and exon 1 HTT protein is greater in Q140 than *Hdh*Q150 brains at 2 months of age. (a) *Htt* intron 1 sequences detected by qPCR (347f‐431r) in the striatum, cortex and cerebellum of Q140 and *Hdh*Q150 mice, and their respective WT littermates at 2 months of age. The level of the *Httexon1* transcript was greater in Q140 than in *Hdh*Q150 mice in all brain regions Student’s *t* test (two‐tailed) ± *SEM*. Intron 1 sequences were not detected in WT mice. (b) Full‐length *Htt* levels as detected by qPCR (‐19f‐ex2r) in the striatum, cortex, and cerebellum of Q140 and *Hdh*Q150 mice as compared to their respective WT littermates at 2 months of age. The level of the correctly spliced *Htt* transcript was lower in the *Hdh*Q150 striatum and cortex compared to WT and there was a trend toward a reduction in the Q140 mice for these two brain regions (one‐way ANOVA with Bonferroni post hoc tests) mean ± *SEM*. (a, b) Q140 mutant (*n* = 5), Q140 WT (*n* = 7), *Hdh*Q150 mutant (*n* = 6), *Hdh*Q150 WT (*n* = 7). Transcripts were normalized to the geometric mean of the housekeeping genes: *Atp5b* and *Eif4a2*. (c) Mutant HTT proteins were immunoprecipitated with the 3B5H10 antibody using Protein G agarose beads (Invitrogen) from either WT, *Hdh*Q150 or Q140 brains at 2 months of age, and western blots were immunoprobed with either MW1 or MW8. MW1 detects the characteristic pattern of N‐terminal HTT fragments (Landles et al., 2010) and MW8 shows that the smallest fragment is the exon 1 HTT protein (arrows). The level of exon 1 HTT is greater in Q140 than in *Hdh*Q150 brains. The smaller fragments migrate at different rates due to variation in the length of the polyQ tract. HTT proteins were immunoprecipitated from *Hdh*Q150 and Q140 brains at 12 months of age. The soluble exon 1 HTT protein is no longer detectable in these lysates, because it has been recruited into aggregates. Immunoprecipitation with mouse IgG antibody alone served as a negative control

We measured the level of intron 1 sequences, using a qPCR assay that amplified sequences located in the 5′ region of *Htt* intron 1, before the first cryptic polyadenylation site. RNA was prepared from striatum, cortex, and cerebellum from 2 month old homozygous Q140 and *Hdh*Q150 mice, together with their respective WT littermates. We detected higher levels of intron 1 sequences in Q140 brains as compared to *Hdh*Q150 in all three brain regions (Figure 4a). The level was between 2.5‐ and 3‐fold higher in the striatum, between 1.5‐ and 2‐fold higher in the cortex, and approximately 1.3‐fold higher in the cerebellum. Incomplete splicing would be expected to lead to lower levels of the correctly spliced, full‐length mutant *Htt* mRNA. In order to determine whether this was the case, we used a qPCR assay that spanned the exon 1—exon 2 splice junction. The level of the spliced mRNA was statistically significantly decreased in the striatum and cortex in *Hdh*Q150 mice, and there was a trend toward a reduction in the Q140 mice in both of these brain regions, when compared to their WT littermates (Figure 4b),

We used the 3B5H10 antibody to immunoprecipitate HTT from the brains of heterozygous Q140 and *Hdh*Q150 mice at 2 and 12 months of age. Heterozygous mice were chosen for this purpose because in homozygous mice, the difference in polyQ length on the mutant HTT alleles makes it difficult to interpret the patterns of N‐terminal fragments immunoprecipitated from their brains. The 3B5H10 antibody recognizes an expanded polyQ repeat and so does not immunoprecipitate HTT from WT tissues (Figure 4c). In contrast, immunodetection with MW1 showed that full‐length HTT, as well as the characteristic pattern of N‐terminal fragments (Landles et al., 2010), were immunoprecipitated from both Q140 and *Hdh*Q150 brains. Comparison of fragment intensities in the immunoprecipitates from the brains of both mouse models at 2 months of age, did not demonstrate any obvious differences for fragments running at ~70 kDa and above. However, the intensity of the smallest fragment that had migrated to the bottom of the gel appeared stronger in the Q140 than in the *Hdh*Q150 lysates. The 3B5H10 immunoprecipitates were resolved on a separate gel, and this time were immunoprobed with MW8, which, on western blots, acts as a neoepitope antibody for the C‐terminus of the exon 1 HTT protein (Landles et al., 2010). MW8 only recognized the smallest fragment, demonstrating that this is exon 1 HTT and that its level is higher in lysates from Q140 brains as compared to *Hdh*Q150 in 2 month old mice (Figure 4c). The exon 1 HTT protein was not present in the 12 month Q140 or *Hdh*Q150 lysates, as by this age it has been recruited into aggregates.

### 3.5 The presence of a neomycin cassette in the Q140 line is unlikely to influence the levels of incomplete splicing

**Figure 5:**
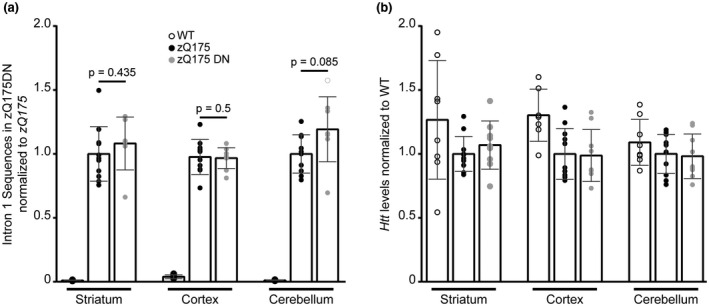
*Httexon1* and full‐length *Htt* transcript levels are comparable in 2 month old zQ175 and zQ175DN brains. (a) *Htt* intron 1 sequences detected by qPCR (347f‐431r) in the striatum, cortex and cerebellum of heterozygous zQ175, heterozygous zQ175DN and WT mice at 2 month of age. There was no difference in the level of the *Httexon1* transcript between zQ175 and zQ175DN mice for all brain regions Student’s *t* test (two‐tailed) mean ± *SEM*. Intron 1 sequences were not detected in WT mice. (b) Full‐length *Htt* levels as detected by qPCR (‐19f‐ex2r) in the striatum, cortex and cerebellum of zQ175, zQ175DN and WT mice at 2 months of age. There was no statistical difference between the groups for any of the brain regions analyzed (one‐way ANOVA with Bonferroni post hoc tests). (a and b) zQ175 (*n* = 12), zQ175DN (*n* = 10), WT (*n* = 8). Transcripts were normalized to the geometric mean of the housekeeping genes: *Atp5b*, *Eif4a2* and *Ubc* (striatum and cerebellum), or *Atp5b*, *Canx* and *Rpl13a* (cortex)

In engineering the Q140 mice to express a chimeric *Htt* gene with human exon 1 and an expanded CAG repeat, a neomycin resistance gene remained located 1.3 kb 5′ to the ATG. Although this neo gene was transcribed in the opposite direction to *Htt*, it is possible that its presence might influence the level of incomplete splicing in the Q140 mice. A spontaneous CAG repeat expansion in the Q140 line has led to the knock‐in mouse line now dubbed zQ175 (Heikkinen et al., 2012; Menalled et al., 2012), from which the neomycin cassette has subsequently been removed to generate the zQ175DN (delta neo) line. In order to investigate the effect of the neo gene on exon 1 splicing, we therefore compared the level of incomplete splicing in 2 month old zQ175 mice, with and without the neomycin cassette. The levels of 5′ intron 1 sequence, before the first cryptic polyA site, were measured by qPCR in the striatum, cortex, and cerebellum (Figure 5a). There was no difference in the levels of these intronic sequences between the zQ175 and zQ175DN mice in any of these brain regions. Therefore, the presence of the neomycin cassette in the Q140 mice was unlikely to be responsible for the increased level of incomplete splicing when compared to that in the *Hdh*Q150 line. The levels of the correctly spliced *Htt* transcripts were also measured by qPCR. Once again, there was no difference in the levels of full‐length *Htt* between the zQ175 and zQ175DN mice in any of the three brain regions tested (Figure 5b).

## 4 DISCUSSION

Previous studies had suggested that the onset of behavioral deficits in Q140 mice might occur much earlier than in *Hdh*Q150 mice (Brooks et al., 2012; Dorner et al., 2007; Heng et al., 2007; Hickey et al., 2008; Lerner, Trejo Martinez Ldel, Zhu, Chesselet, & Hickey, 2012; Lin et al., 2001; Menalled et al., 2003; Rising et al., 2011; Simmons et al., 2009; Woodman et al., 2007). To systematically evaluate these potential differences, we standardized the genetic background by generating Q140 and *Hdh*Q150 C57BL/6J congenic lines, bred them to homozygosity and maintained them under identical husbandry conditions. We performed carefully controlled comparisons of behavioral phenotypes, and confirmed that, as we had previously shown, the Q140 mice exhibited deficits in exploratory behavior in the open field at 1, 4, and 6 months of age, spontaneous alternation in the Y‐maze at 3 months, the vertical pole test at 4 months and night time wheel running performance at 6 months. However, the *Hdh*Q150 mice had yet to develop a deficit in any of these tests by 6 months of age. Although the CAG repeat expansions in these two lines were relatively well‐matched, they were slightly higher in the *Hdh*150 mice, and so could not have accounted for these differences.

The deposition of aggregated HTT in the striatum precedes that in most other brain regions in both the Q140 (Menalled et al., 2003) and *Hdh*Q150 knock‐in lines (Woodman et al., 2007). Within neuronal nuclei, immunohistochemistry first reveals this as a diffuse staining pattern that fills the entire nucleus, from which puncta appear and then coalesce into a single large nuclear inclusion as pathogenesis proceeds (Li et al., 1999; Menalled et al., 2003). We know that this diffuse staining pattern represents an aggregated form of HTT, because it cannot be detected by immunohistochemistry with antibodies that recognize the polyQ tract, as this epitope is buried in the aggregate structure; however, pretreatment with formic acid, breaks open the cross‐β‐sheet H‐bonding, exposing the polyQ epitope (Landles et al., 2010). In order to determine whether the deposition of HTT aggregates in the striatum occurs at different ages in Q140 and *Hdh*Q150 mice, we performed immunohistochemistry with MW8, an antibody for which the AEEPLHRP epitope is present in both mouse and human HTT, and therefore, in both of these mouse lines. At 4.5 months of age, immunostaining with MW8 revealed a prominent neuronal nuclear staining pattern that was barely detectable in striatal sections from *Hdh*Q150 mice, indicating that the aggregation process has started earlier in the Q140 striata. To investigate whether transcriptional dysregulation might also initiate at different ages, we measured the levels of a signature set of striatal transcripts, known to be dysregulated in HD mice, at 6.5 months of age. We found that these transcripts were down‐regulated in both lines to comparable extents, indicating that they could not contribute the behavioral deficits in the Q140 mice, as these had not been detected in the *Hdh*Q150 line. It would be necessary to measure these transcripts at an earlier age in order to determine whether, as with aggregation, transcriptional dysregulation occurs earlier in Q140 mice.

We had previously shown that all knock‐in mouse lines express the highly pathogenic exon 1 HTT protein, through the incomplete splicing of exon 1 *Htt* to exon 2 (Sathasivam et al., 2013). Therefore, the earlier onset of the behavioral phenotypes in the Q140 line might have occurred because they expressed higher levels of exon 1 HTT. To investigate this possibility, we measured the levels of 5’ intron 1 *Htt* sequences in the striatum, cortex and cerebellum in homozygous Q140 and *Hdh*Q150 mice at 2 months of age. We found that the levels of the *Httexon1* mRNA were 2–3‐fold higher in the striatum, 1.5–2‐fold higher in the cortex and ~1.3‐fold higher in the cerebellum in CAG140 as compared to *Hdh*Q150 mice. Immunoprecipitation of mutant HTT from whole brain lysates, followed by western blotting, indicated that the Q140 mice had higher levels of the exon 1 HTT protein, as would be predicted. Consistent with the occurrence of incomplete *Htt* splicing, the levels of spliced *Htt* were decreased in both lines when compared to WT levels.

**Figure 6:**
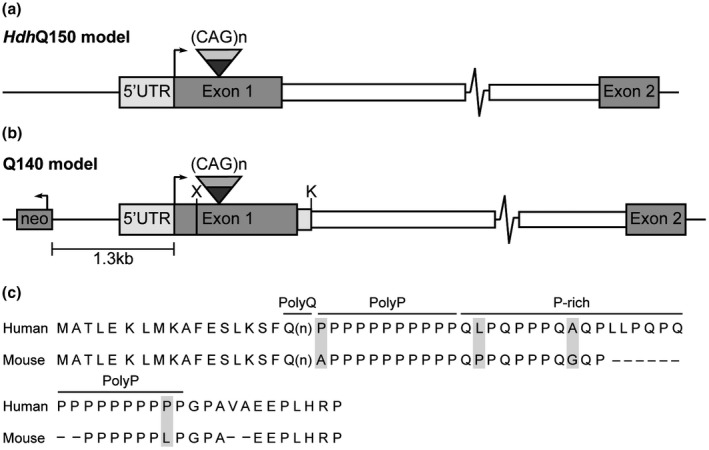
Genomic structures and protein sequence of the genetically modified *Htt* locus of Q140 and *Hdh*Q150 knock‐in mice. (a and b) Schematics of the mutant *Htt* loci in the *Hdh*Q150 and Q140 knock‐in mice. (a) In the *Hdh*Q150 model, the mouse (CAG)_2_CAA(CAG)_4_ sequence has been replaced by an expanded CAG repeat by a double homologous recombination event that has left no other sequence modifications (Lin et al., 2001). (b) In the Q140 model, the XmnI—KpnI mouse *Htt* sequences have been replaced with the human XmnI—KpnI fragment (Levine et al., 1999). This left the amino acid sequence of the first 17 amino acids before the polyQ repeat unchanged, but the amino acid sequence following the polyQ differs between human and mouse HTT. In addition, 84 bp at the 5′ end of mouse intron 1 have been replaced with 10 bp from the 5′ end of human intron 1 and an intact neomycin resistance gene cassette (*neo*) located 1.3 kb 5′ to the initiation codon. X = XmnI, K = KpnI. (c) Alignment of the amino acid sequence of human and mouse exon 1 HTT. The first 17 amino acids before the polyQ repeat are identical in both species. The polyQ tract in human HTT is followed by a polyproline (polyP) repeat, a 17 amino acid proline‐rich (P‐rich) domain, a second polyP repeat and then 12 amino acids that terminate in a proline residue. In comparison, mouse HTT has a 6 amino acid deletion in the P‐rich domain (LLPQPQ), the second polyP repeat contains 6 rather than 10 residues, a dipeptide VA deletion in the 12 C‐terminal amino acids, as well as four substitutions (shown in gray). Neo = neomycin

The strategy by which the Q140 and *Hdh*Q150 knock‐in lines were created resulted in a number of sequence differences in the 5’ region of the modified *Htt* genes (Figure 6). In the *Hdh*Q150 mice an expanded CAG repeat had been introduced into mouse *Htt* through a double homologous recombination replacement event that resulted in no other modifications to the locus (Lin et al., 2001) (Figure 6a). In contrast, the Q140 mice were generated by replacing an XmnI—KpnI fragment of mouse *Htt* with an XmnI—Kpn from human *HTT* containing an expanded CAG repeat (Levine et al., 1999; Menalled et al., 2003) (Figure 6b). Therefore, Q140 and *Hdh*Q150 mice differ in the human and mouse exon 1 HTT amino acid sequences (Figure 6c), and the Q140 mice also have a neomycin gene cassette 5’ to the ATG and 84 bp of mouse intron 1 has been replaced with 10 bp of human intron 1. The zQ175 line has arisen through a spontaneous CAG repeat expansion in the Q140 line (Menalled et al., 2012), and zQ175 and Q140 mice carry the same modifications to the 5′ end of the *Htt* gene. We were able to show that the level of incomplete splicing did not differ between zQ175 and zQ175DN, in which the neomycin gene had been removed, and therefore the presence of the neo was unlikely to underlie the differences in splicing efficiency between the Q140 and *Hdh*Q150 lines that we have identified in this study. We have shown that the splicing factor SRSF6, which recognizes a CAG repeat motif, binds to mutant exon 1 mRNA, and we propose that this may favor the incomplete splicing of exon 1 to exon2 through the ectopic recruitment of the U1RNP spliceosome complex (Neueder, Dumas, Benjamin, & Bates, 2018). Incomplete splicing could be higher in the Q140 line through structural or proximity effects brought about by human exon 1, as compared to mouse exon 1 sequences. Alternatively, splicing enhancers that are located in the 5′ region of intron 1 might have been deleted in the Q140 line.

The exon 1 HTT protein has been shown to be highly pathogenic in a wide range of cellular and *in vivo* HD models (Barbaro et al., 2015; Mangiarini et al., 1996) and is known to aggregate readily (Scherzinger et al., 1997). Of these, R6/2 mice are transgenic for a genomic fragment that spans the 5′ end of the human *HTT* gene and contains promoter sequences, *Htt* exon 1 and some intron 1 sequences (Mangiarini et al., 1996). The transgene is transcribed from the human promoter, through the human exonic and intronic sequences and terminates after transcribing into mouse sequence at the integration site. Therefore, R6/2 mice are a model of the incomplete splicing that occurs in all knock‐in models of HD. There are multiple studies that show that late‐stage disease phenotypes that develop in R6/2 and knock‐in mice are highly comparable (Kuhn et al., 2007; Labbadia et al., 2011; Mielcarek et al., 2014, 2015; Moffitt, McPhail, Woodman, Hobbs, & Bates, 2009; Woodman et al., 2007), suggesting that exon 1 HTT contributes to the pathogenic process in these knock‐in models. Our data suggest that earlier age of onset in Q140 as compared to *Hdh*Q150 mice, for the sensorimotor and pathological phenotypes that we have assessed, may be caused by the increased levels of exon 1 HTT. However, our data do not rule out the contribution of other factors, for example, the differences between the mouse and human polyproline regions of exon 1 present in *Hdh*Q150 and Q140, although functional consequences of these sequence differences at the protein level have not been identified, and levels of full length mutant HTT have been reported to decrease more slowly with age in *Hdh*Q150 than in Q140 mice (Franich et al., 2018). Nevertheless, our data suggest that targeting the production and/ or levels of the *Htt*exon*1* transcript, presents a rational approach to complement existing therapeutic strategies.

## DECLARATION OF TRANSPARENCY

The authors, reviewers, and editors affirm that in accordance to the policies set by the Journal of Neuroscience Research, this manuscript presents an accurate and transparent account of the study being reported and that all critical details describing the methods and results are present.

## CONFLICT OF INTEREST

The authors have no conflict of interest to report.

## AUTHOR CONTRIBUTIONS

All authors had full access to all the data in the study and take responsibility for the integrity of the data and the accuracy of the data analysis. *Conceptualization*, N.R.F., M.A.H., C.Z., D.H., G.P.B., and M.F.C.; *Methodology*, N.R.F., M.A.H., C.Z., A.N., C.L., and D.H.; *Investigation*, N.R.F., M.A.H, C.Z, G.F.O, N.A., T.C., N.H.B, V.L., R.P.L., A.N., and C.L.; *Formal Analysis*, N.R.F, M.A.H., C.Z., N.A., G.F.O., A.N., and C.L.; *Resources*, S.Z.O. and D.H.; *Writing Original Draft*, N.R.F., G.P.B., and M.F.C.; *Writing – Review & Editing*, N.R.F., C. Z., G.P.B., and M.F.C.; *Visualization*, N.R.F., C. Z., G.F.O., and C.L.; *Supervision*, A. N., G. P. B., and M.F.C.; *Funding Acquisition*, G.P.B. and M.F.C.

## Supporting information

[Correction added on July 30, 2019 after first online publication: The heading ‘Transparent Peer Review Report.’ was added and Peer review communication document was uploaded online.]

Transparent Science Questionnaire for Authors.

Transparent Peer Review Report.

## Data Availability

The data that support the findings of this study are available from the corresponding authors upon reasonable request.
