# Supplementary material for: Phenotype onset in Huntington’s disease knock‐in mice is correlated with the incomplete splicing of the mutant huntingtin gene
Source: J Neurosci Res. 2019 Jul 7;97(12):1590–605. doi: 10.1002/jnr.24493 (PMC6801054; doi:10.1002/jnr.24493)
Supplement: Supplementary file 2 — Transparent Peer Review Report. [file JNR-97-1590-s002.docx]

**Phenotype severity in Huntington’s disease knock-in mice is associated with incomplete splicing of the mutant huntingtin gene**

Nicholas R. Franich, Miriam A. Hickey, Chunni Zhu, Georgina F. Osborne, Nadira Ali, Tiffany Chu, Nicholas H. Bove, Vincent Lemesre, Renata P. Lerner, Scott O. Zeitlin, David Howland, Andreas Neueder, Christian Landles, Gillian P. Bates and Marie-Francoise Chesselet

_____________________________________________________________________________________

| Review timeline: | Submission date: 27/03/19  Editorial Decision: Minor Modification  Revision Received: 21/05/19  Accepted: 17/06/19 |  |
| --- | --- | --- |
|  |  |  |

Editor: Carlos Cepeda

Reviewers: Joshua Barry, Diane Merry

_____________________________________________________________________________________

1st Editorial Decision	29/04/19

_____________________________________________________________________________________

29-Apr-2019 

Dear Dr Bates: 

Thank you for submitting your manuscript to the Journal of Neuroscience Research. We've now received the reviewer feedback and have appended those reviews below. I'm glad to say that the reviewers are overall very enthusiastic and supportive of the study. They did raise some concerns and made some suggestions for clarification, but I expect that these points should be relatively straightforward to address. If there are any questions or points that are problematic, please feel free to contact me. I am glad to discuss. 

We ask that you return your manuscript within 30 days. Please explain in your cover letter how you have changed the present version. If you require longer than 30 days to make the revisions, please contact Dr. Eric Prager (eprager@wiley.com). You can submit your revised manuscript directly by clicking on the following link: *** PLEASE NOTE: This is a two-step process. After clicking on the link, you will be directed to a webpage to confirm. *** 

https://mc.manuscriptcentral.com/jnr?URL_MASK=28a017b601f3436d875694581ab6e0e3 

Thank you again for your submission to the Journal of Neuroscience Research; we look forward to reading your revised manuscript. 

Best Wishes, 

Dr Carlos Cepeda 
Associate Editor, Journal of Neuroscience Research 

Dr Eric Prager 
Editor-in-Chief, Journal of Neuroscience Research 

Reviewer: 1 

Comments to the Author 
The authors of this paper examined how phenotype onset and rate of disease progression varies between two Huntington’s disease (HD) mouse models, the Q140 and the HdhQ150. The authors used behavioral tests, Western blots and immunohistochemistry to show that mutant hutingtin aggregation appears earlier in the striatum of Q140 than HdhQ150 mice. This suggests that the aggregation of the mutant huntingtin contributes to the severity of the disease phenotype which is seen earlier in the Q140 versus the HdhQ150 mouse model. 

This paper was well organized and all experiments were necessary and sufficient to support the authors assertion that the length of the HD mutation influences the disease phenotype in the two mouse models studied. The immunohistochemistry (Fig 2) and Western blots (Fig 4) nicely show an increase in the mutant huntingtin aggregates both in brain slices and overall protein amount in the striatum, respectively. 


Reviewer: 2 

Comments to the Author 
Manuscript #jnr-2019-Mar-7999 by Franich et al., entitled “Phenotype severity in Huntington’s disease knock-in mice is associated with incomplete splicing of the mutant huntingtin gene,” describes robust behavioral, neuropathological and biochemical studies of Q140 and HdhQ150 knock-in mouse models of Huntington’s disease in an effort to illuminate the role of incomplete splicing of huntingtin exon 1 and the production of an exon 1-encoding mutant Huntingtin protein fragment in the different disease onset ages in these two mouse models.  The thorough evaluation is robust and compelling in defining the different disease onset in these two models; however, the conclusions about the role of incomplete splicing in the different disease onset ages are correlative and should be more tempered.  This issue and additional issues are listed below, and should be considered. 

1. It is not only possible but likely that the different exercise on the running wheel impacts the transcriptional output in the two mouse models.  Thus, this caveat should be considered when making the conclusion that transcriptional defects in the two lines are the same.  More importantly, see point #2. 

2. The conclusion in Section 3.3 that homozygous Q140 and HdhQ150 mice have similar levels of transcriptional dysregulation at 6.5 months of age and that, given the different ages of onset in these two mouse models, the transcriptional repression of Drd2, Penk1, Darpp32 or Cnr1 does not influence the onset of behavioral phenotypes in these lines, is flawed.  First, the transcriptional dysregulation does not appear to be similar – the decreases appear to be more substantial in the Q140 mice than in the HdhQ150 mice. Furthermore, unless the authors analyze the transcription of these genes prior to the onset of symptoms for the Q140 mice, I don't see how they can conclude that they do not influence the onset of behavioral phenotypes. It could be that they are more modestly decreased at 3 weeks in the Q140 mice and not at all in the HdhQ150 mice at this age. 

3. A minor point about the aggregate analysis with antibody MW8.  Just because the epitope is present in both human and mouse sequences doesn't mean that it detects both epitopes with equal efficiency.  There are other sequence differences in exon 1 that could impact the folding and, thus, the availability of this epitope in the two lines. Is there evidence in the literature or from the author’s work that the antibody detects the mouse and human proteins with equal affinity? 

4. The penultimate sentence of the discussion, which addresses the possibility that other sequence differences between mouse and human exon 1 could contribute to the different disease onset in Q140 and HdhQ150 mice, is insufficient.  Overall, the conclusions of the authors are too strong for the data presented.  The title and the last two sentences of the abstract should be edited, and additional discussion of this point within the discussion should be added.  As for the title, although the wording is technically true, that phenotype onset (not severity) is “associated” (meaning “correlated”) with incomplete splicing, it is also “associated” with sequence differences in exon 1. Stating the former while omitting the latter is misleading, in the absence of additional discussion. 

5. Significance statement:  The statement that these results provide insights into the mechanism of incomplete splicing is incorrect.  There do not appear to be data in this manuscript that address the mechanism of incomplete splicing, unless it is to suggest that differences in mouse vs. human sequences impact the extent of incomplete splicing.

____________________________________________________________________________________

Authors’ Response	21/05/2019

_____________________________________________________________________________________

Reviewer: 1

Comments to the Author

The authors of this paper examined how phenotype onset and rate of disease progression varies between two Huntington’s disease (HD) mouse models, the Q140 and the HdhQ150. The authors used behavioral tests, Western blots and immunohistochemistry to show that mutant huntingtin aggregation appears earlier in the striatum of Q140 than HdhQ150 mice. This suggests that the aggregation of the mutant huntingtin contributes to the severity of the disease phenotype which is seen earlier in the Q140 versus the HdhQ150 mouse model.

This paper was well organized and all experiments were necessary and sufficient to support the authors assertion that the length of the HD mutation influences the disease phenotype in the two mouse models studied. The immunohistochemistry (Fig 2) and Western blots (Fig 4) nicely show an increase in the mutant huntingtin aggregates both in brain slices and overall protein amount in the striatum, respectively.

We thank the reviewer for their positive and supportive comments.

Reviewer: 2

Comments to the Author

Manuscript #jnr-2019-Mar-7999 by Franich et al., entitled “Phenotype severity in Huntington’s disease knock-in mice is associated with incomplete splicing of the mutant huntingtin gene,” describes robust behavioral, neuropathological and biochemical studies of Q140 and HdhQ150 knock-in mouse models of Huntington’s disease in an effort to illuminate the role of incomplete splicing of huntingtin exon 1 and the production of an exon 1-encoding mutant Huntingtin protein fragment in the different disease onset ages in these two mouse models. The thorough evaluation is robust and compelling in defining the different disease onset in these two models; however, the conclusions about the role of incomplete splicing in the different disease onset ages are correlative and should be more tempered. This issue and additional issues are listed below, and should be considered.

We thank the reviewer for their positive comments

1.	It is not only possible but likely that the different exercise on the running wheel impacts the transcriptional output in the two mouse models. Thus, this caveat should be considered when making the conclusion that transcriptional defects in the two lines are the same. More importantly, see point #2.

In our study, both the Q140 and HdhQ150 mice have been housed in identical conditions, for 6 months, and then exposed to the same running wheel task. In a previous report, environmental enrichment, over a period of 24 weeks, did not affect the level of transcriptional dysregulation for the striatal genes that we have measured, when mice housed in an enriched environment were compared to those without enrichment (Benn et al. 2010 J Neuropath. Exp. Neurol. 69, 817-827).

If there were any subtle effects on transcriptional read outs caused by the differences in running wheel performance between the two mouse lines, these were not detectable. The striatal genes are dysregulated in the HdhQ150 mice, despite the absence of a motoric phenotype and the consequential comparatively better running wheel performance. We have added a sentence to discuss this point toward the end of the first paragraph on page 19.

2.	The conclusion in Section 3.3 that homozygous Q140 and HdhQ150 mice have similar levels of transcriptional dysregulation at 6.5 months of age and that, given the different ages of onset in these two mouse models, the transcriptional repression of Drd2, Penk1, Darpp32 or Cnr1 does not influence the onset of behavioral phenotypes in these lines, is flawed. First, the transcriptional dysregulation does not appear to be similar – the decreases appear to be more substantial in the Q140 mice than in the HdhQ150 mice. Furthermore, unless the authors analyze the transcription of these genes prior to the onset of symptoms for the Q140 mice, I don't see how they can conclude that they do not influence the onset of behavioral phenotypes. It could be that they are more modestly decreased at 3 weeks in the Q140 mice and not at all in the HdhQ150 mice at this age.

We show that at 6.5 months of age, specific striatal transcripts are dysregulated to comparable levels in the Q140 and HdhQ150 mice. The Q140 mice have a motor phenotype, whereas the HdhQ150 mice do not. If these dysregulated striatal transcripts were driving the motor phenotypes that we have measured, the HdhQ150 mice should also be impaired.

Our one way ANOVA and post hoc statistical analyses did not detect a statistically significant difference in the level of transcriptional dysregulation between the Q140 and HdhQ150 lines.

We had already included the following sentence at the end of the first paragraph on page 23 of the discussion:

‘It would be necessary to measure these transcripts at an earlier age in order to determine whether, as with aggregation, transcriptional dysregulation occurs earlier in the Q140 mice.

3.	A minor point about the aggregate analysis with antibody MW8. Just because the epitope is present in both human and mouse sequences doesn't mean that it detects both epitopes with equal efficiency. There are other sequence differences in exon 1 that could impact the folding and, thus, the availability of this epitope in the two lines. Is there evidence in the literature or from the author’s work that the antibody detects the mouse and human proteins with equal affinity?

Unlike S830 or EM48, which do detect mouse and human exon 1 HTT differently, we have no evidence that this is the case for MW8, and have added a comment on this at the end of the first paragraph on page 18. However, a definitive proof would not be easy. We would need a model system, preferably mouse, in which human exon 1 HTT, and mouse exon 1 HTT are expressed at the same levels, with the CAG repeat expansions equivalent to those in the knock-in mice used in this study. It would be necessary to demonstrate comparable detection of both soluble and aggregated HTT. Any other approach would be limited by caveats.

4.	The penultimate sentence of the discussion, which addresses the possibility that other sequence differences between mouse and human exon 1 could contribute to the different disease onset in Q140 and HdhQ150 mice, is insufficient. Overall, the conclusions of the authors are too strong for the data presented. The title and the last two sentences of the abstract should be edited, and additional discussion of this point within the discussion should be added. As for the title, although the wording is technically true, that phenotype onset (not severity) is “associated” (meaning “correlated”) with incomplete splicing, it is also “associated” with sequence differences in exon 1. Stating the former while omitting the latter is misleading, in the absence of additional discussion.

The reviewer is correct and we have changed ‘severity’ in the title to ‘onset’ and ‘associated’ to ‘correlated’.

The fact that the sequence differences in the huntingtin gene, RNA or protein lead to the differences in phenotype cannot be disputed. It is the functional consequences of these sequence differences that need to be identified.

We have found that the sequence differences at the RNA level lead to a difference in the levels of incomplete splicing and the consequential production of the exon 1 HTT protein, which over the past 20 years, has been shown to be highly pathogenic in many model systems.

The main difference in the protein sequences is highlighted in Figure 6. There is an additional 8 amino acids (LLPQPQP) in the human proline-rich region that is located between the two proline repeats. Whilst this might have functional consequences, there are none that have been demonstrated to date, and therefore, we are not able to elaborate on this. We have added this point to the discussion toward the end of the first paragraph on page 25.

We have cited the previous Franich paper showing that the levels of full-length HTT decline with age at different rates in these two models, although these data need to be confirmed by more quantitative approaches.

We have rewritten the abstract to give a more balanced perspective. We have left the last sentence in place, as given the current interest from Pharma and the CHDI Foundation, no-one disagrees with this.

5.	Significance statement: The statement that these results provide insights into the mechanism of incomplete splicing is incorrect. There do not appear to be data in this manuscript that address the mechanism of incomplete splicing, unless it is to suggest that differences in mouse vs. human sequences impact the extent of incomplete splicing.

The referee is correct and we have removed the statement about mechanism from the significance statement.
